# Reduction in C-Peptide Levels and Influence on Pharmacokinetics and Pharmacodynamics of Insulin Preparations: How to Conduct a High-Quality Euglycemic Clamp Study

**DOI:** 10.3389/fphar.2021.786613

**Published:** 2021-12-02

**Authors:** Yi Tao, Mingxue Zhu, Junliang Pu, Peilin Zhang, Lei Wan, Chengyong Tang

**Affiliations:** Department of Phase I Clinical Trial Ward, the First Affiliated Hospital of Chongqing Medical University, Chongqing, China

**Keywords:** euglycemic clamp, C-peptide, glucose oscillation, pharmacodynamics, pharmacokinetics

## Abstract

**Objective:** The aim of the study was to investigate the different extent of inhibition of endogenous insulin secretion by the reduction of C-peptide levels in an euglycemic clamp study and its effects on the evaluation of pharmacokinetics, pharmacodynamics of insulin preparations, and quality of clamp study to determine the best reduction range of C-peptide levels.

**Methods:** Healthy Chinese male volunteers were enrolled and underwent a single-dose euglycemic clamp test. Participants were subcutaneously injected with long-acting insulin glargine (0.4 IU/kg). Blood samples were collected pretest and up to 24 h post-test to assess pharmacokinetics (PK), pharmacodynamics (PD), and C-peptide levels.

**Results:** We divided the 39 volunteers enrolled in the study into three groups according to the reduction of C-peptide levels: group A (ratio of C-peptide reduction <30%, *n* = 13), group B (ratio of C-peptide reduction between ≥ 30% and <50%, *n* = 15), and group C (ratio of C-peptide reduction ≥50%, *n* = 11); there were significant differences in the three groups (*p* **=** 0.000). The upper and lower limits of blood glucose oscillation in group C was statistically lower than the other groups, the range of oscillating glucose levels in group C was −17.0 ± 6.6% to −1.1 ± 6.7%. The AUC_0–24 h_ in groups A, B, and C were 9.7 ± 2.2, 11.0 ± 2.9, and 11.9 ± 2.1 ng/ml × min, respectively, which indicated an increasing trend in the three groups (*P*_trend_ = 0.041). For quality assessment, the average glucose (*p* = 0.000) and MEFTG (*p* = 0.001) levels in three groups were significantly different.

**Conclusion:** The different extent of inhibition of endogenous insulin will influence the PK/PD of insulin preparations and the quality of the euglycemic clamp. Furthermore, the ratio of C-peptide reduction should be above 50% to free from the interference of endogenous insulin, and the range of blood glucose levels should be consistently maintained at −10% to 0 in the euglycemic clamp.

## Introduction

Considering the endogenous insulin and glucose self-regulatory mechanisms, how to precisely evaluate the pharmacokinetics (PK) and pharmacodynamics (PD) of insulin preparations has always been a challenge. According to the EMA, the endogenous insulin production of health volunteers can influence PK and PD measurements (European Medicines Agency, 2015). Thus, it is extremely important to suppress endogenous insulin during clamping study. Four primary techniques have been extensively applied: 1) the hyperinsulinemic-euglycemic clamp establishes a higher plasma level insulin plateau by continuous intravenous insulin infusion (e.g., 1.0 mU/kg/min) (DeFronzo et al., 1979; Bokemark et al., 2000; James et al., 2020); 2) the low-level insulin-infusion euglycemic clamp allows continuous intravenous insulin infusion below the basal level (e.g., 0.10–0.15 mU/min/kg) to suppress endogenous insulin secretion (Starke et al., 1989; Heinemann et al., 1999; Cernea et al., 2004); 3) the somatostatin-infused euglycemic clamp establishes an euinsulinemic-euglycemic clamp by infusing somatostatin to inhibit endogenous insulin secretion (Mergler et al., 2008; Heise et al., 2016); and 4) the euglycemic clamp without exogenous insulin inhibits endogenous insulin by clamping blood glucose levels below the subject’s fasting glucose level (Scholtz et al., 2005; Sørensen et al., 2010; Heise et al., 2015a; Linnebjerg et al., 2015).

The hyperinsulinemic-euglycemic clamp technique has been widely used in previous studies, although it overestimates the effects of insulin preparations, especially long-acting preparations (Soop et al., 2000; Kim, 2009; James et al., 2020). The euglycemic clamp without exogenous insulin is a method of controlling blood glucose levels below the subject’s fasting glucose to suppress endogenous insulin secretion. A previous study using both techniques evaluating the pharmacokinetics of insulin preparations has indicated that artificially established non-physiological hyperinsulinemia interferes with the PK/PD parameters of insulin preparations (Liu et al., 2019). However, the euglycemic clamp without exogenous insulin was superior, which is currently used to evaluate the PK/PD of insulin preparations.

C-peptide is a polypeptide originating from proinsulin, which releases insulin and C-peptide in equimolar amounts into the circulation (Yaribeygi et al., 2019). However, before reaching the peripheral circulation, the liver extracts a part of insulin (approximately 50%), while only a minority of C-peptide is extracted. Moreover, the half-life of the C-peptide is longer than that of insulin (20–30 min vs. 3–5 min) (Hoekstra et al., 1982; Jones and Hattersley, 2013). In conclusion, peripheral C-peptide is a more appropriate and accurate marker for assessing endogenous insulin secretion (Alieva et al., 1985; Jones and Hattersley, 2013). A previous study indicated that a C-peptide suppression of over 50% would have been more powerful for indicating adequate restriction of endogenous insulin (Liu et al., 2021).

Blood glucose oscillations should be controlled within a specific range by infusion of glucose. According to the EMA, in healthy subjects, the target blood glucose value should be set below the subjects fasting glucose (e.g., 0.3 mmol/L or 10%); the closer the blood glucose concentration to the target, the more successful the clamp is in achieving its goal of maintaining the desired glycemic plateau (Benesch et al., 2015; European Medicines Agency, 2015). However, the blood glucose concentration is oscillating if it is unclamped, which will result in insufficient suppression of endogenous insulin and affect the accuracy of pharmacodynamics data (Benesch et al., 2015; Heise et al., 2016). There are some quality assessment indices used in glucose clamping such as the coefficient of variation in blood glucose (CVBG), percentage of the glucose excursion from target range (GEFTR), and the mean excursion from target glucose (MEFTG) (Benesch et al., 2015). The EMA suggests that the calculation of mean values, root mean square deviation, and CV of blood glucose concentrations should be provided to estimate the performance of the clamp study (European Medicines Agency, 2015). Controlling of glucose oscillations is of great importance to inhibit endogenous insulin secretion (Benesch et al., 2015). However, it is unknown whether a different degree of inhibition of endogenous insulin secretion is associated with glucose oscillations, PK/PD assessment, and the quality of clamp study.

In this study, healthy male volunteers were enrolled and underwent a 24-h euglycemic clamp study. Our objective was to investigate the different extent of inhibition of endogenous insulin secretion, which is reflected by the ratio of C-peptide reduction, and its effects on the PK and PD of long-acting insulin preparations, thus determining the best reduction range of C-peptide and exploring the way to improve the quality of clamp study, which could provide a theoretical basis for future empirical research.

## Materials and Methods

### Research Design

The volunteers were screened and qualified for the entry standard. A 24-h euglycemic glucose clamp study was conducted in healthy male subjects after 0.4 iu/kg insulin glargine injection. During the trial, volunteers were requested to avoid strenuous exercises, smoking, drinking alcohol, or caffeinated drinks (e.g., tea and coffee). In order to investigate the best reduction extent of C-peptide, we divided the subjects into three groups according to the ratio of C-peptide reduction by using the empirical and quantile classification method, based on the values of 33.3% quantile, that is, group A: C-peptide reduction rate <30%; group B: C-peptide reduction rate between ≥30% and <50%; and group C: C-peptide reduction rate ≥50%. The trial was carried out in accordance with the principles of the Declaration of Helsinki. This study was approved by the Ethics Committee of the First Affiliated Hospital of ChongQing Medical University (No. 20190101).

### Subjects

Healthy Chinese male volunteers aged 18–45 years were enrolled. We selected individuals with a body mass index (BMI) of 19–24 kg/m^2^ without diabetes, insulin resistance, or a family history of diabetes, who did not have insulin resistance, and who did not have cardiovascular disease. The volunteers were non-smokers and non-alcohol users. Subjects had no abnormalities on routine OGTT (oral glucose tolerance test), insulin releasing test (IRT), blood and urine examinations, liver and kidney function tests, and electrocardiograms. All volunteers provided written informed consent prior to the start of the study.

### Euglycemic Clamp Procedures

All recruited volunteers underwent a single-dose euglycemic clamp test. Participants arrived at the ward on the day prior to the clamp test to ensure a 10-h fasting condition and to maintain fasting during euglycemic clamping. Insulin glargine was injected into a lifted abdominal skinfold (0.4 IU/kg). Intravenous access was obtained in one arm for a 20% glucose infusion and in the other for blood drawing. The arm for blood drawing was heated using a warming blanket to arterialize venous blood (55–65°C). Blood samples were collected before dosing and up to 24 h post-dosing to analyze glargine and C-peptide levels at the following time points: −30 min, −20 min, −10 min, and 0 min, and 0.5 1, 2, 3, 4, 5, 6, 8, 10, 12, 15, 18, 21, and 24 h. PD variables were evaluated during euglycemic clamping lasting up to 24 h, which included blood samples drawn for biochemical analysis at 10-min intervals at 30 min before injection and up to 8 h after injection, at 20-min intervals from 8 to 16 h, and at 30-min intervals from 16 to 24 h.

Basal glucose (BG) was defined as the average blood glucose level before injection; the target glucose (TG) level was defined as the BG minus 0.28 mmol/L (Heise et al., 2012; Heise et al., 2015b); and we controlled the blood glucose concentration as close as possible to the TG level. The glucose infusion rate (GIR) was manually adjusted by investigators to maintain blood glucose at the target value. The euglycemic clamp was used to inhibit endogenous insulin secretion and to maintain blood glucose concentrations constant; the GIR profile over time represented the activity of the insulin preparations.

### Bioanalytical Methods

Blood glucose concentration was immediately analyzed using an automatic glucose oxidase analyzer Biosen C-line GP+(Germany) during clamping, whose qualification range was 0.5–50 mmol/L. The glargine and C-peptide samples were transferred to Covance Laboratories after processing and centrifugation. Insulin glargine is rapidly metabolized to its active metabolites M1 and M2; therefore, the glargine prototype drug and the concentrations of the metabolites M1 and M2 were used for pharmacokinetic analysis, which were evaluated by a validated liquid chromatography–tandem mass spectrometry (LC-MS/MS), whose qualification range was 0.07–2.5 ng/ml. The C-peptide concentration was analyzed by ELISA, the qualification range of which was 20–3,000 pmol/L.

### Statistical Methods

SPSS 22.0 and WinNonlin 8.1 were used for statistical analysis. C-peptide concentration was quantified to monitor endogenous insulin secretion, and the C-peptide reduction rate was calculated as 1-mean CPt/CP0. The oscillation of glucose was calculated with formula 1-Gt (glucose at time t)/Gd (desired glucose). Parameter estimates were computed by non-compartmental analysis (NCA) of the total insulin glargine concentration versus time profiles and glucose infusion rate versus time profiles, and the pharmacokinetic parameters were area under the glargine concentration versus time curve (AUC_0–24_), peak glargine concentration (C_max_), and time to C_max_ (T_max_). The pharmacodynamic parameters were area under the glucose infusion rate versus time curve (AUCGIR_0–24 h_), peak of glucose infusion rate (GIR_max_), and time to GIR_max_ (TGIR_max_).

For quality evaluation indicators, CVBG was calculated as the SD of the blood glucose/mean value of blood glucose. The GEFTR was calculated as the degree of glucose excursion from target range/total blood glucose at specific time points. The MEFTG was calculated as the mean excursion of target glucose.

Quantitative data were expressed as mean ± standard deviation (SD) or median values with interquartile ranges (25–75%). Normality was examined, and some data were natural log transformed prior to analysis. Statistical analysis was performed using the analysis of variance (ANOVA) or the Kruskal–Wallis test. The relationship between the ratio of C-peptide reduction and blood glucose was assessed using Pearson correlation coefficients. *p* < 0.05 (two sided) was considered statistically significant.

## Results

### Demographics and Clinical Characteristics

Thirty-nine volunteers were enrolled after screening for eligibility. The demographics and clinical characteristics of the subjects in the three groups are summarized in Table 1. The mean ± SD of the ratio of C-peptide reduction was 39 ± 14%. There were significant differences in the ratio of C-peptide reduction among the groups (Table 1).

**TABLE 1:** Demographics and clinical characteristics of the subjects.

	Group A (*n* = 13)	Group B (*n* = 15)	Group C (*n* = 11)	*p*-value
Age (y)	26.9 ± 5.2	29.3 ± 5.9	26.0 ± 4.0	0.238
BMI (kg/m^2^)	22.5 ± 1.2	22.0 ± 1.0	21.9 ± 1.5	0.346
SBP (mmHg)	120.2 ± 10.2	123.1 ± 9.4	117.7 ± 7.2	0.346
DBP (mmHg)	72.0 ± 8.7	78.6 ± 7.5	73.7 ± 6.8	0.078
HR (times/min)	73.2 ± 10.8	75.5 ± 11.7	72.7 ± 9.1	0.77
Fasting serum insulin (mmol/L)	4.4 ± 2.3	4.3 ± 4.5	4.8 ± 3.5	0.887
Fasting glucose (mmol/L)	5.3 ± 0.3	5.3 ± 0.3	5.0 ± 0.3	0.093
HOMA-IR	0.9 ± 0.7	1.1 ± 1.3	1.0 ± 1.1	0.736
Dose (IU)	25.7 ± 2.4	25.4 ± 1.6	25.1 ± 2.3	0.783
Ratio of C-peptide reduction (%)	22.7 ± 5.4	39.8 ± 5.3	55.6 ± 3.9	0.000

Data are expressed as mean ± SD or medians (25–75%). Statistical analysis was performed using analysis of variance (ANOVA) or the Kruskal–Wallis test, *p* < 0.05.

Volunteers were 27.6 ± 5.2 years old and had a BMI of 22.1 ± 1.2. There were no significant differences in the prevalence of risk factors for metabolic syndrome or cardiometabolic disease, including BMI, SBP (systolic blood pressure), DBP (diastolic blood pressure), HR (heart rate), fasting serum insulin, fasting glucose, and HOMA-IR. The doses of glargine in groups A, B, and C were 25.7 ± 2.4, 25.4 ± 1.6, and 25.1 ± 2.3 IU, respectively; there were no significant differences in administered doses (*p* = 0.783).

### C-Peptide Profiles

**FIGURE 1:**
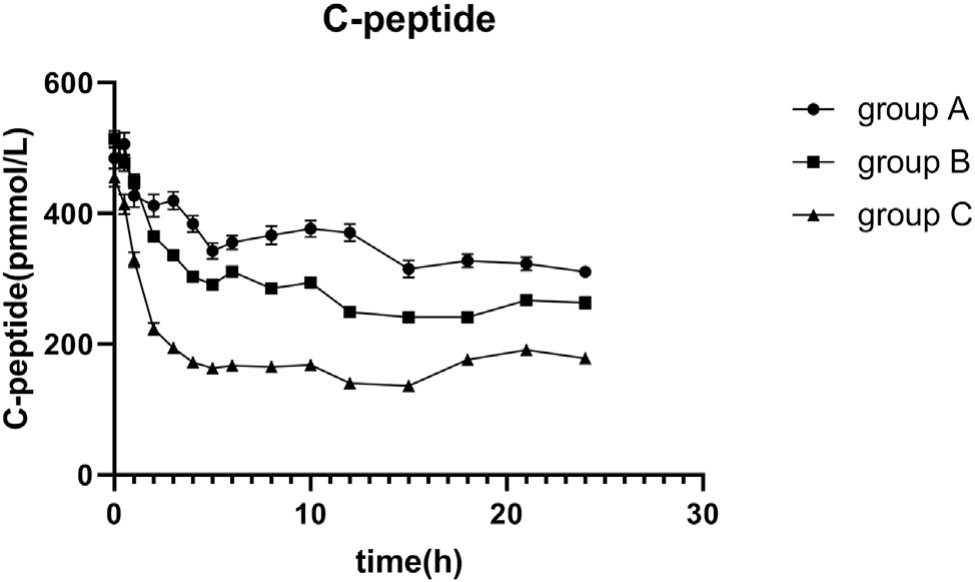
Mean C-peptide concentration versus time profiles after 0.4 IU/kg doses of glargine insulin in healthy volunteers. The error bars represent the 95% confidence intervals. The extent of C-peptide reduction in group C was apparently higher than that in the others, and there were statistically significant differences among groups.

Endogenous insulin secretion was restrained by euglycemic clamps, and the serum C-peptide levels were used to reflect the degree of restriction. The basal C-peptide levels were 484.6 ± 207.1 pmol/L in group A, 514.0 ± 184.4 pmol/L in group B, and 455.4 ± 154.6 pmol/L in group C. The profiles of C-peptide changes over time were shown in Figure 1; C-peptide showed a descending trend in all groups, which means that the endogenous insulin secretion was restrained to different degrees. Results showed that there were significant differences in the ratio of C-peptides reduction among the three groups (*p* = 0.000).

### Variations in PD and PK Parameters During Euglycemic Clamping

**FIGURE 2:**
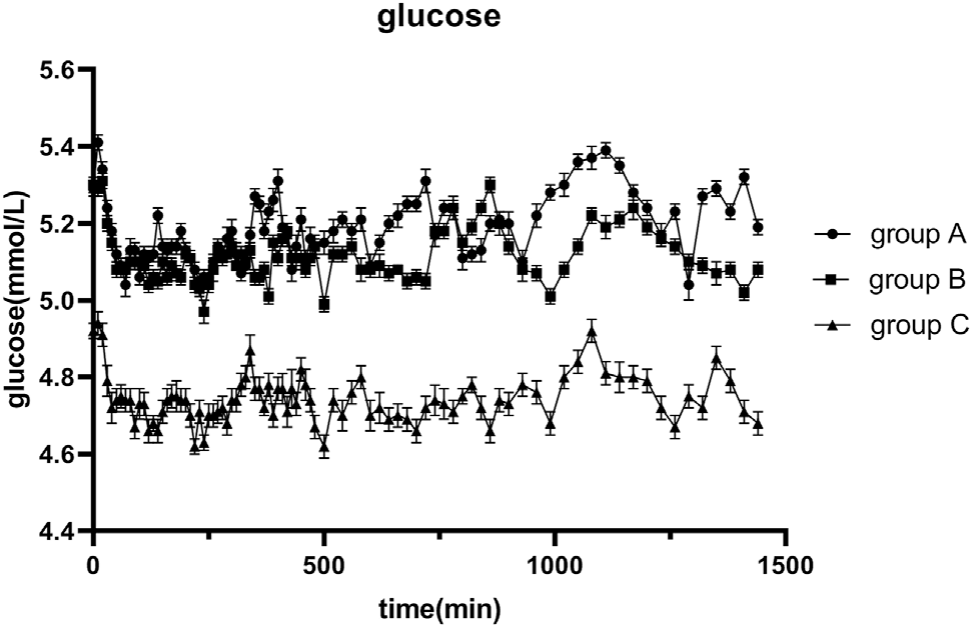
Blood glucose versus time after 0.4 IU/kg doses of glargine insulin in healthy volunteers. The error bars represent the 95% confidence intervals. The blood glucose in group C was obviously lower than that in the others, and significances were found in three groups.

**FIGURE 3:**
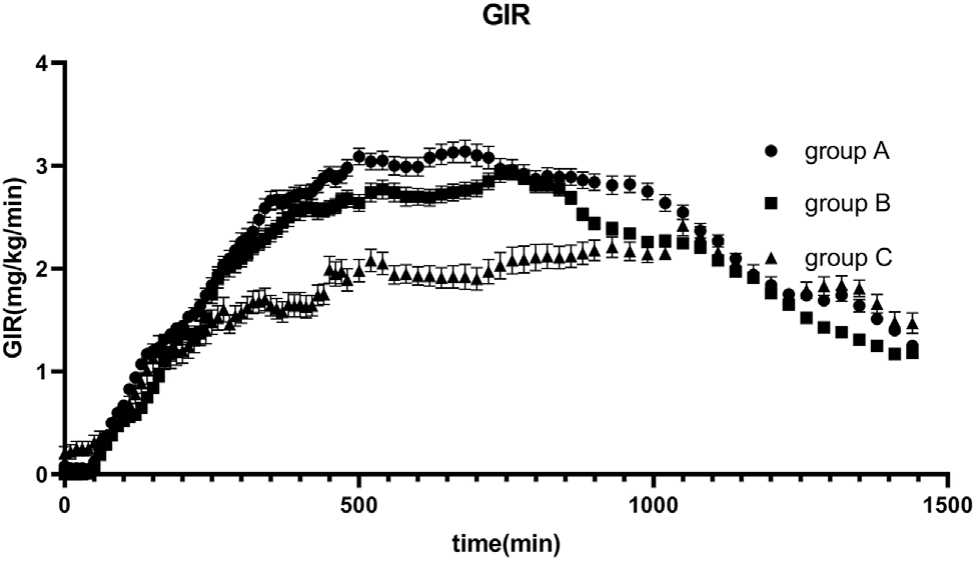
Glucose infusion rate (GIR) versus time profiles after 0.4 IU/kg doses of glargine insulin in healthy volunteers. The error bars represent the 95% confidence intervals. GIR in group C was lower than that in the others, but there were no statistical differences among groups.

**FIGURE 4:**
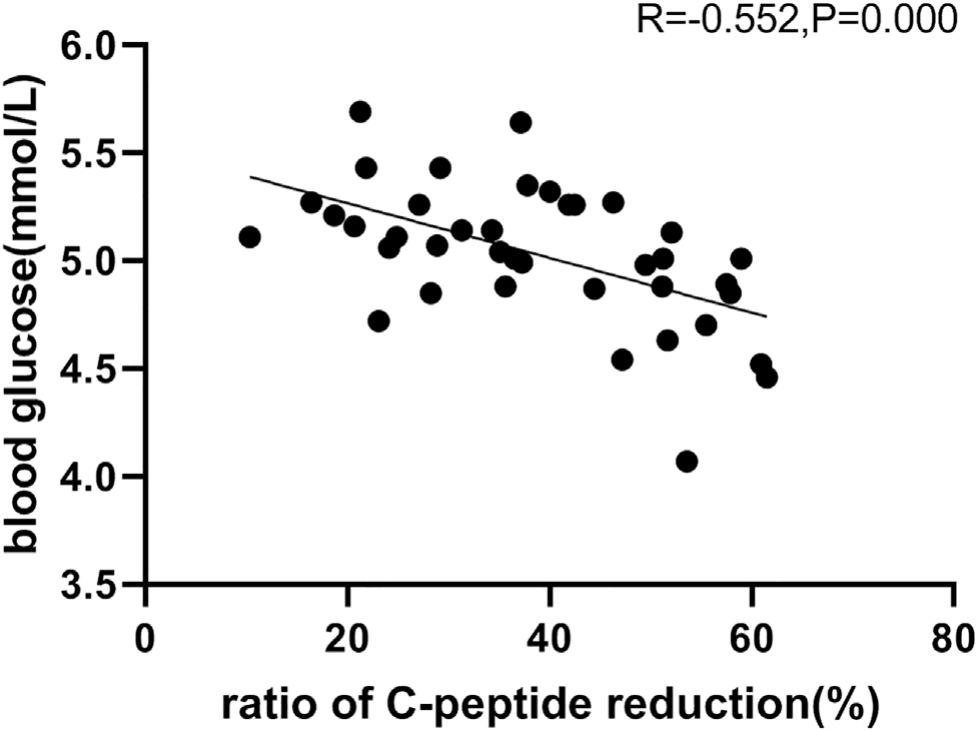
Relationship between the ratio of C-peptide reduction and blood glucose, in which revealed a negative correlation.

The average glucose target level achieved in groups A, B, and C were 4.99, 4.98, and 4.90 mmol/L, respectively. The GIR was adjusted in accordance with glucose values during the clamping procedure. The profiles of the oscillations of glucose and GIR over time were shown in Figures 2, 3. According to the figures, it was obvious that group C achieved lower glucose levels and had a lower glucose infusion rate than groups A and B. The ratio of C-peptide reduction was related to blood glucose (*r* = −0.552 *p* = 0.000, Figure 4). The oscillation ranges of glucose in all groups were reported in Table 2, ranges of which were [−10.7 ± 7.9%, 10.2 ± 5.5%] in group A [−11.4 ± 6.3%, 7.1 ± 5.1%] in group B, and [−17.0 ± 6.6%, −1.1 ± 6.7%] in group C; there were significant differences in group C compared to the others. As shown in Table 3, although there was no significant difference in PD parameters, the GIR_max_ in group C was obviously lower than that in the other groups; TGIR_max_ in group C showed an increase, which was more prolonged than that in the other groups; and AUCGIR_0–24 h_ in group C was lower than that in the other groups.

**TABLE 2:** Oscillating range of blood glucose.

	Group A	Group B	Group C	*p-value*	*p* trend
Lower limit (%)	−10.7 ± 7.9	−11.4 ± 6.3	−17.0 ± 6.6	0.070	0.035
Upper limit (%)	10.2 ± 5.5	7.1 ± 5.1	−1.1 ± 6.7*	0.000	0.000

Data are expressed as means ± SD, *p*＜0.05. “*” means differences between group C and groups A and B.

**TABLE 3:** Pharmacokinetics (PK) and pharmacodynamics (PD) parameters in three groups.

	Group A	Group B	Group C	*p-value*	*p* trend
PD					
GIR_max_ (mg/kg/min)	3.5 ± 1.3	3.4 ± 1.4	3.0 ± 1.5	0.586	0.326
T_GIRmax_ (min)	593.1 ± 198.1	584.0 ± 199.1	756.4 ± 228.0	0.088	0.062
AUC_GIR0–24 h_ (mg/kg)	3175.9 ± 1135.2	2862.9 ± 1176.0	2526.1 ± 1222.0	0.411	0.186
PK					
C_max_ (ng/ml)	0.6 ± 0.2	0.6 ± 0.2	0.7 ± 0.2	0.293	0.120
T_max_ (h)	11.7 ± 4.0	10.4 ± 3.1	12.8 ± 2.2	0.182	0.403
AUC_0–24 h_ (ng/ml × min)	9.7 ± 2.2	11.0 ± 2.9	11.9 ± 2.1	0.112	0.041

Data are expressed as means ± SD, *p <* 0.05.

**FIGURE 5:**
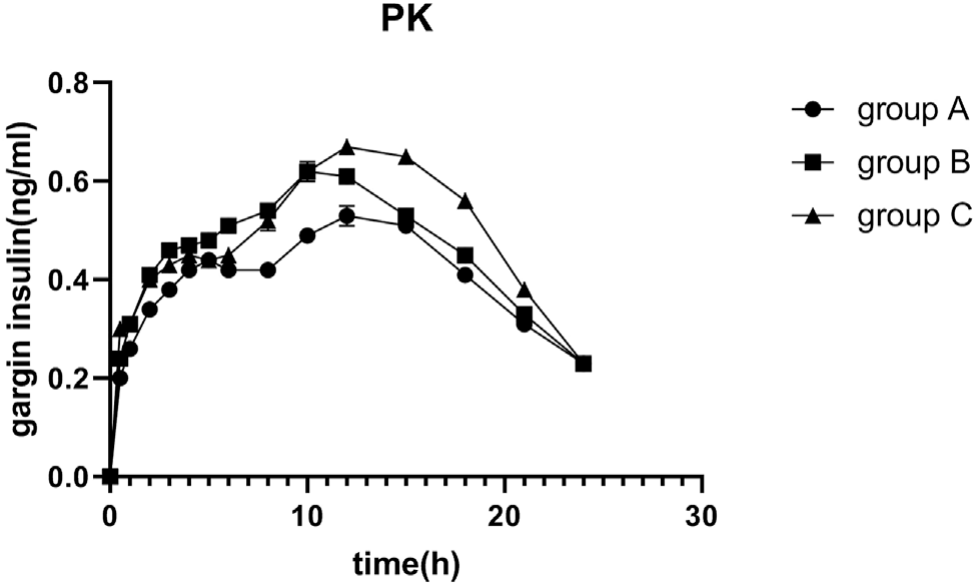
Mean insulin glargine concentration versus time profiles after 0.4 IU/kg doses of glargine insulin in healthy volunteers. The error bars represent the 95% confidence intervals. Glargine concentration was higher than that in the others, but there were no statistical differences among groups.

The profile of plasma glargine insulin concentrations following the 0.4 IU/kg injection over time was shown in Figure 5, and the curves fitted well. There were no significant differences in the PK parameters; however, T_max_ and AUC_0–24 h_ in group C were higher than those in the other groups. Furthermore, AUC_0–24 h_ revealed an increasing trend in three groups (P_trend_ = 0.041).

### Indexes of Euglycemic Clamp Quality Assessment

We evaluated the quality of the euglycemic clamp by assessing mean glucose levels, SD, CVBG, MEFTG, and GEFTR (Table 4). There was a significant difference in average glucose levels across the three groups (*p* = 0.000): Group C had lower mean glucose than groups A and B, revealing a decreasing trend in all groups (P_trend_ = 0.000). MEFTG was significantly different across all three groups (*p* = 0.001), and the mean excursion of target glucose was lower in groups A and B than in group C, which revealed a decreasing trend in groups (P_trend_ = 0.000). Although there were no statistical differences in CVBG and GEFTR, the values of group C were markedly lower than those of the other groups (Table 4).

**TABLE 4:** Indexes of the quality assessment of euglycemic clamp in three groups.

	Group A	Group B	Group C	*p*-value	*p* trend
Average glucose (mmol)/l	5.2	5.1	4.8	0.000**	0.000
standard deviation SD (mmol/l)	0.2	0.2	0.2	0.148	0.053
CVBG (%)	4.4 ± 1.5	3.7 ± 1.2	3.6 ± 0.6	0.182	0.097
MEFTG (mmol/l)	−0.01 ± 0.25	−0.08 ± 0.26	−0.45 ± 0.31	0.001*	0.000
GEFTR (%)	2.1 ± 3.2	1.8 ± 2.8	1.0 ± 1.3	0.592	0.316

Data are expressed as means ± SD; SD of average glucose was listed separately, *p <* 0.05. “*” means differences between group C and groups A and B. “**” means differences in three groups.

### Safety Evaluation

There were nine AEs observed in six subjects. Mild elevation of serum bilirubin (*N* = 3), urine ketone body positive (*N* = 3), urinary protein positive (*N* = 1), elevation of creatinine (*N* = 1), and hypokalemia (*N* = 1) were observed with no action required. No notable hypoglycemia, allergic reaction, and adverse reactions at the injection site were observed.

## Discussion

Insulin analogs and their biosimilars are commonly used for the treatment of patients with diabetes; therefore, their safety and efficacy should be noticed (Owens et al., 2012). The euglycemic clamp, an acknowledged method for evaluating PK/PD of insulin preparations, should be applied before new insulin preparations, and their analogs are launched into the market (Østerberg et al., 2003; Becker et al., 2011). The key of the euglycemic clamp is to maintain glucose within a certain range by regulating exogenous glucose infusion (Heise et al., 2016). We manually adjusted exogenous glucose infusion rates according to target glucose levels to ensure a higher clamp quality (Heinemann and Ampudia-Blasco, 1994; Home, 2015); however, there were some factors that affected timely glucose adjustment, such as individual metabolic differences, delayed blood drawing time, and infusion pump variability, which involved a delay in converting the infusion rate in milliliters per minute to the correct final dial setting (DeFronzo et al., 1979; Bequette, 2009; Heise et al., 2016). These factors increased the difficulty of maintenance of glucose levels. If the blood glucose level sustained above the baseline level, this will lead to increased endogenous insulin secretion, which will overestimate the PD properties of the insulin preparation. Hence, adequate inhibition of endogenous insulin secretion is crucial during the euglycemic clamp procedure, which could be monitored by the reduction in C-peptide after dosing (Liljenquist et al., 1978; DeFronzo et al., 1979; Kaga et al., 2019). PK and PD are the essential indices for evaluation of insulin preparation, and furthermore, clamp quality assessment is of great importance (Benesch et al., 2015). In this study, we divided volunteers into three groups according to the ratio of C-peptide reduction; explored whether the different extent of inhibition of endogenous insulin would influence PK and PD assessment and the quality of the euglycemic clamp; and determined the superior range of glucose regulating.

Healthy volunteers were enrolled in this study, who presented homogenous characteristics and insulin sensitivity. Healthy volunteers exhibit lower intra-individual variability than patients with type 1 diabetes mellitus (T1DM). Furthermore, insulin secretion in women may vary during the menstrual cycle, although it is unclear whether this may influence study results (European Medicines Agency, 2015). According to the EMA guidelines, commonly used insulin doses in the clamp study should range from 0.3 to 0.4 IU/kg bodyweight for intermediate-acting insulin (European Medicines Agency, 2015), and considering previous studies and instruction on glargine administration (Heise et al., 2012; Heise et al., 2015a; Bhatia et al., 2018), we decided to use a dose of 0.4 IU/kg.

Endogenous insulin secretion should be strictly controlled by the euglycemic clamp technique as endogenous insulin secretion interferes with PK and PD properties (Porcellati et al., 2015). Endogenous insulin and C-peptide are also released from islet β-cells; therefore, the C-peptide could be applied as a marker to measure the suppression of endogenous insulin secretion (Alieva et al., 1985; Jones and Hattersley, 2013; Leighton et al., 2017; Yaribeygi et al., 2019). We observed that the C-peptide in the three groups fluctuated below the baseline levels, which indicated that endogenous insulin was restricted in all three groups. However, the ratio of C-peptide reduction was significantly decreased in the three groups.

Factors that influence clamping include the metabolic activity of the subject, sensitivity to insulin, the dosage of the insulin preparation, and glucose regulation during clamping (Porcellati et al., 2015). We recruited volunteers with strict entry criteria. Results displayed that there were no differences in demographics, clinical characteristics, and dosage levels achieved in the three groups, which indicated that demographics and clinical characteristics would not affect the euglycemic clamp test when the volunteers strictly adhered to the enrollment criteria.

The results indicated that the ratio of C-peptide reduction in group C is the highest; thus, the contribution from the secretion of endogenous insulin was inhibited more thoroughly, and the PD could best reflect the real effect of exogenous administered insulin. According to the oscillating range of glucose, the ceiling and floor limits of glucose oscillation in group C were significantly lower than those in the other groups. In order to control the coefficient of variation, based on the results of this study, we suggest that the glucose regulating range should be maintained in −10% to 0. GIR_max_ and AUC_GIR0–24 h_ in group C were lower than those in the other groups, and T_GIRmax_ was more prolonged in group C than in the other groups. Although there were no significant differences in the PD parameters, values differed across the three groups. For PK parameters, C_max_, T_max_, and AUC_0–24 h_ in group C were higher than those in the other groups. Thus, the extent of the reduction in the C-peptide influences the assessment of PD and PK properties, and adequate suppression of endogenous insulin secretion is crucial for successful clamping. Consequently, we suggest that the ratio of C-peptide reduction should be higher than 50%, with which the clamp study immunes to the disturbance of endogenous insulin, and this conclusion was consistent with previous studies (Bhatia et al., 2018).

There is currently no gold standard for evaluating euglycemic quality; the CVBG is commonly used as a quality indicator in euglycemic clamp studies, and a CVBG value ≤ 5% is considered superior (Heinemann and Ampudia-Blasco, 1994). However, the CVBG only reflects blood glucose oscillations, and the accuracy of maintaining glucose level at a specific target is ignored. In this study, we comprehensively evaluated mean glucose levels, SD, CVBG, MEFTG, and GEFTR as quality evaluation indicators (Benesch et al., 2015; European Medicines Agency, 2015). In the light of our findings, group C was superior to the other groups, indicating that different inhibition extent of endogenous insulin could influence the quality of the euglycemic clamp study: the better the inhibition of endogenous insulin, that is, the greater the reduction of C-peptide, the better the clamp quality is. Consequently, the level of C-peptide reduction should be recommended as a quality assessment indicator.

## Conclusion

Based on our findings, we propose that C-peptide levels should be below those of baseline values during the clamp, and the extent of the reduction in C-peptide levels will influence the PK/PD of insulin preparations and the quality of euglycemic clamps. Furthermore, the C-peptide reduction ratio should be greater than 50% to ensure better inhibition of endogenous insulin secretion. For glucose regulation, the oscillating glucose ranging from −10% to 0 is recommended. Finally, the ratio of C-peptide reduction should be considered a quality evaluation indicator of euglycemic clamp tests.

## Data Availability

The original contributions presented in the study are included in the article/supplementary material; further inquiries can be directed to the corresponding author.
